# Comparison between Different Extraction Methods for Determination of Primary Aromatic Amines in Food Simulant

**DOI:** 10.1155/2018/1651629

**Published:** 2018-05-15

**Authors:** Morteza Shahrestani, Mohammad Saber Tehrani, Shahram Shoeibi, Parviz Aberoomand Azar, Syed Waqif Husain

**Affiliations:** ^1^Department of Analytical Chemistry, Faculty of Basic Science, Science and Research Branch, Islamic Azad University, Tehran, Iran; ^2^Food and Drug Laboratories Research Center (FDLRC), Iran Food and Drug Administration (IFDA), MOH, Tehran, Iran; ^3^Department of Food Chemistry, Food and Drug Laboratories Research Center (FDLRC), Iran Food and Drug Administration (IFDA), MOH, Tehran, Iran; ^4^Department of Chemistry, Faculty of Science, Science and Research Branch, Islamic Azad University, Tehran, Iran; ^5^Department of Analytical Chemistry, Faculty of Basic Sciences, Azad University, Sciences and Researches Branch, P.O. Box 14515-775, Poonak-Hesarak, Tehran, Iran

## Abstract

The primary aromatic amines (PAAs) are food contaminants which may exist in packaged food. Polyurethane (PU) adhesives which are used in flexible packaging are the main source of PAAs. It is the unreacted diisocyanates which in fact migrate to foodstuff and then hydrolyze to PAAs. These PAAs include toluenediamines (TDAs) and methylenedianilines (MDAs), and the selected PAAs were 2,4-TDA, 2,6-TDA, 4,4′-MDA, 2,4′-MDA, and 2,2′-MDA. PAAs have genotoxic, carcinogenic, and allergenic effects. In this study, extraction methods were applied on a 3% acetic acid as food simulant which was spiked with the PAAs under study. Extraction methods were liquid-liquid extraction (LLE), dispersive liquid-liquid microextraction (DLLME), and solid-phase extraction (SPE) with C18 ec (octadecyl), HR-P (styrene/divinylbenzene), and SCX (strong cationic exchange) cartridges. Extracted samples were detected and analyzed by HPLC-UV. In comparison between methods, recovery rate of SCX cartridge showed the best adsorption, up to 91% for polar PAAs (TDAs and MDAs). The interested PAAs are polar and relatively soluble in water, so a cartridge with cationic exchange properties has the best absorption and consequently the best recoveries.

## 1. Introduction

Primary aromatic amines (PAAs) are food contaminants. Migration of PAAs into food is through colored plastics, printed paper, cooking utensils, and flexible packaging. One of the main sources of PAAs is polyurethane (PU) adhesives which are used extensively in lamination of multilayer films. PU adhesives might contain unreacted aromatic diisocyanates coming from the imperfect polymerization process of polyurethane; after packaging, water present in foods hydrolyzes residual aromatic diisocyanates, thus leading to PAA formation [1].

Toluene diisocyanate (TDI) and methylene diphenyl isocyanate (MDI) are used in the production of PU adhesives. TDIs are often a mixture (80 : 20) of the two isomers: 2,4-TDI and 2,6-TDI, while MDIs consist of a mixture of higher oligomer homologues, 4,4′-MDI (40–50%), 2,4′-MDI (2.5–4.0%), and 2,2′-MDI (0.1–0.2%) [2].

PAAs have genotoxic, carcinogenic, and allergenic effects. Epidemiological studies of the 4,4′-MDA indicate a risk of bladder cancer in humans, and the 2,4-TDA and 4,4′-MDA are listed as possible human carcinogens [3].

In the European Union (EU) Regulation (10/2011), plastic material and articles shall not release PAAs in detectable quantity (DL = 0.01 mg/kg) in food or food simulants. Chromatography method is recommended for individual identification and quantification of migrated PAAs [4].

The extraction/preconcentration of PAAs is required in order to reach the detection limit, prior to analysis. Sample preparation is commonly done for LLE or SPE method [5]. Each one of these two general approaches has advantages and disadvantages; also there has been a new development in the two methods in order to save time, labor, chemicals, and other materials used. This process has led to the creation of new methods such as solid-phase microextraction (SPME), liquid-phase microextraction (LPME), and dispersive liquid-liquid microextraction (DLLME) [6].

DLLME technique has been developed and often used for the determination of organic compound in water samples. DLLME is a very simple and rapid extraction method which is used for preconcentration of organic and inorganic compounds from aqueous samples. This method is based on the fast injection of the appropriate mixture of extraction (high-density solvent) and dispersive solvents (polar) into the aqueous solution to form a cloudy ternary component solvent. Extraction process is being completed with centrifugation step, and the enriched analyte is collected in the shape of droplets in the sediment phase [7–9].

Several methods are provided in the literatures for determination of TDIs and MDIs or TDAs and MDAs in the urine, blood, PU foam, water, and waste water samples. These methods are often used with or without hydrolysis of samples, extraction/preconcentration in case of liquid-liquid extraction (LLE) or solid-phase extraction (SPE), and with or without derivatization for GC or HPLC analysis. The selection of extraction methods depends on the type and condition of the sample [10–14].

In this study, two different methods of liquid-phase extraction (LLE, DLLME) and solid-phase extraction (Chromabond C18 ec, Chromabond HR-P, and SCX) were investigated for the extraction of interested PAAs, which were spiked in a 3% acetic acid as food simulant [15]. Use of 3% acetic acid which is accepted by the FDA offers the best condition for simulation and a worst-case scenario for this study, and also it is easy to evaluate [16, 17]. The selection of aromatic amines was based on the type of diisocyanates used to produce PU adhesives. The polyurethane adhesives are often produced from toluene diisocyanates (2,4-TDI and 2,6-TDI) or methylene diphenyl isocyanate (4,4′-MDI); thus, the PAAs presented are 2,4-TDA, 2,6-TDA, 4,4′-MDA, 2,4′-MDA, and 2,2′-MDA, and the relative recoveries of the PAAs of interest are determined in the 3% acetic acid matrix.

## 2. Materials and Methods

### 2.1. Standards and Reagents

The reagents used in this work were all of analytical grade with no further purification. All the reagents were obtained from Merck KGaA, Darmstadt, Germany.

2,6-Toluenediamine, 2,4-toluenediamine, and 4,4′-methylenedianiline (purity > 98%) were obtained from Sigma-Aldrich, and 2,4′-methylenedianiline and 2,2′-methylenedianiline (purity > 95%) were from Angene.

### 2.2. Apparatus and Conditions

HPLC system was an Agilent 1200, equipped with a quaternary pump (G1311A), a column thermostat (G1316A), a degasser unit (G1322A) an autosampler (G1329), and a diode array detector (G1315D). The HPLC system was controlled, and data were analyzed by a computer equipped with LC software (Agilent Chem Station).

The chromatographic conditions were as follows: HPLC analysis was performed with methanol (solvent A) and an ammonium acetate buffer 10 mM (solvent B) as mobile phase. Separation with solvent programming was accomplished on a Phenomenex C18 column (250 × 4.6 mm i.d., 5 *μ*m particle sizes); flow rate was 1 ml·min^−1^, detection wavelength was 235 nm, and temperature was adjusted to 25°C. The gradient elution process was performed as follows in order to achieve the optimum separation: solvent A was kept at 15% and solvent B was kept at 85% for 2 min, solvent A percentage was increased to 65% along a linear gradient curve for 24 min, and then, solvent A concentration was again increased from 65% to 100% along a linear gradient curve for further 2 min and kept steady at 100% for another 2 min and then was decreased to 15% in the same manner in 1 min and continued for a further 2 min, giving the total run time of 30 min.

### 2.3. Standard Solutions and Sample Solutions

The individual stock solutions of each standard with concentration of 100 mg·l^−1^ in methanol were prepared. These solutions were kept in the darkness and under refrigeration (4°C) for up to six months. Mixed intermediate standard solutions in methanol were prepared by dissolving appropriate amounts of each individual solution to yield concentrations of 10 mg·l^−1^. Calibration solutions in methanol/sodium citrate solution 0.1 M (25/75) were prepared daily in the range of 50–800 ng·ml^−1^.

The sample solutions were prepared from standard mix solutions 10 mg·l^−1^ with three concentrations 7.5, 15, and 30 ng·ml^−1^ in 3% acetic acid (*w*/*v*).

### 2.4. Extraction Methods

#### 2.4.1. Liquid-Liquid Extraction Method

For the extraction of PAAs by solvents, the optimized method was applied [12, 13] and modified as follows: an aliquot sample solution of 8 ml was transferred to a test tube, the pH was adjusted to above 10 with sodium hydroxide, and the solution was saturated with sodium chloride. The sample was extracted three times with 5 mL portion of mixed dichloromethane/petroleum benzene (3 : 1) and followed by twice with 5 mL of mixed dichloromethane/methyl tert-butyl ether (1 : 1). The organic solvent fractions were combined and evaporated to dryness by a stream of nitrogen gas. Finally, the extract was dissolved in 0.8 mL of methanol/sodium citrate solution 0.1 M (25/75) and filtered through a 0.22 *μ*m filter for HPLC analysis.

#### 2.4.2. Dispersive Liquid-Liquid Microextraction Method

The method used in DLLME was obtained from Wang et al. [8] and Zhou et al. [9], which was modified by the description given below.

A volume of 5 mL sample solution was placed in a conical bottom tube, 1 g sodium chloride was added, and the pH was adjusted to 12 using a 5 N NaOH solution. A mixture of 500 *μ*L acetonitrile and 90 *μ*L toluene was rapidly injected into the sample using a syringe, and a cloudy solution was obtained. The solution thus prepared was then shaken for 2 min and was then centrifuged at 6000 rpm for 5 min. The dispersed fine droplets of toluene were sedimented at the bottom of the test tube. The upper layer was removed, and the residual phase was blown to almost dryness with low-pressure nitrogen gas. Finally, the extract was dissolved in 0.5 mL of methanol/sodium citrate solution 0.1 M (25/75) and filtered through a 0.22 *μ*m filter for HPLC analysis.

#### 2.4.3. SPE Chromabond® C18 ec

SPE cartridges were conditioned based on the method explained by Oostdyk et al. [5] as the procedure with 5 ml methanol and 5 ml distillated water. Then, 50 ml sample solution was adjusted to pH 10 with 10 M sodium hydroxide solution and was passed slowly through the cartridges. The cartridges were eluted three times with 1 ml of ethyl acetate. Eluents were evaporated in a stream of nitrogen. The remaining residue was dissolved in 5 mL of methanol/sodium citrate solution 0.1 M (25/75) and collected into a 5 ml graduated tube. The solutions were filtered through a 0.22 *μ*m filter for HPLC analysis.

#### 2.4.4. SPE Chromabond HR-P

SPE cartridges were conditioned as advised by Macherey-Nagel Company [11], with 2 × 5 ml methanol, 2 × 5 ml acetonitrile, and 2 × 5 ml 10^−5^ M sodium hydroxide solution. Then, 50 ml sample solution was adjusted to pH 9 with 10 M sodium hydroxide solution and was passed slowly through the cartridges. The cartridges were eluted by 3 × 1.5 ml methanol/acetonitrile (1 : 1). Eluents were evaporated in a stream of nitrogen. The remaining residue was dissolved in 5 mL of methanol/sodium citrate solution 0.1 M (25/75) and collected into a 5 ml graduated tube. The solutions were filtered through a 0.22 *μ*m filter for HPLC analysis.

#### 2.4.5. SPE SCX

Aznar et al. [16] applied SCX cartridges for PAAs, and the method was modified as below: cartridges were conditioned with 2 × 3 ml methanol and 2 × 3 ml 3% acetic acid (*w*/*v*). Then, 50 ml sample solution was passed slowly through the cartridges. The cartridges were eluted with 5 × 1 ml methanol/sodium citrate solution 0.1 M (25/75) and collected into a 5 ml graduated tube. The solutions were filtered through a 0.22 *μ*m filter for HPLC analysis.

### 2.5. Method Validation of SPE SCX

#### 2.5.1. Linearity

Linearity was evaluated for mix of five PAAs with concentrations of 50, 100, 200, 400, and 800 *μ*gl^−1^ in methanol/sodium citrate solution 0.1 M (25/75) with three replications (*n*=3) (Table 1).

There was a good linearity in the range of 0.05–0.8 mgl^−1^ for all five target compounds.

#### 2.5.2. Accuracy

In order to investigate the recoveries of the method including SPE procedures, blank food simulants (3% acetic acid) spiked with five PAAs at three different concentrations 7.5, 15, and 30 *μ*gl^−1^ were performed with three replications (*n*=3). The recoveries are presented for the five aromatic amines (Table 2).

These data confirm not only the accuracy of the method, but also the integrity of the SPE procedure.

#### 2.5.3. Precision

Precision calculation was done based on the repeatability criterion in one day (intraday) and in 3 consecutive days (interday).

Intraday precision was evaluated with spiked blank food simulant (3% acetic acid) at three different concentrations 7.5, 15, and 30 *μ*gl^−1^ including SPE procedures. The RSD of intraday was obtained (Table 3).

Interday precision was evaluated with spiked blank food simulant (3% acetic acid) at three different concentrations 7.5, 15, and 30 *μ*gl^−1^ including SPE procedures on three different days. The RSD of interday was obtained (Table 3).

#### 2.5.4. Sensitivity

Relative standard deviation (RSD) of the method was performed with spiked blank food simulant (3% acetic acid) by five PAAs at concentration 7.5 *μ*gl^−1^, including the SPE procedure. LOD and LOQ of the method were calculated (Table 4).

## 3. Results and Discussion

The migration of primary aromatic amines (PAAs) from flexible food packaging represents a serious risk to public health as these compounds are potentially carcinogenic substances. The source of PAAs is from the residues of aromatic diisocyanates (2,4-TDI, 2,6-TDI, and 4,4′-MDI) arising from incomplete curing of the main polyurethane (PU) adhesive, and also other aromatic diisocyanates such as 2,4′-MDI and 2,2′-MDI are present in adhesive inappropriately.

The five PAAs (2,4-TDA, 2,6-TD, 4,4′-MDA, 2,4′-MDA, and 2,2′-MDA) used in this study were selected on the basis of their origin of PU adhesives used in food packaging. Toxicity of PAAs was evaluated and classified into three groups based on their levels of toxicity with 2,4-TDA, 2,6-TDA, and 4,4′-MDA being in the high toxicity class [18]. According to the EU Regulation (EU 10/2011), detection limit of the released PAAs must be below 10 *µ*g/kg of food or food simulant. The method was developed and designed based on the worst-case scenario for the migration of PAAs from packaging material into a food simulant which in this study was 3% acetic acid. The aim of the research was to design and select an extraction method, liquid and/or solid phase, of analysis for these PAAs (TDAs and MDAs) with high sensitivity and also to make possible to understand the origin of the PAAs detected. The method was designed for 3% acetic acid (as food simulant) in purified water, since this simulant was considered the most restrictive, that means it uses the worst-case scenario for the migration of TDAs and MDAs from food packaging. The direct analysis of the simulant by HPLC-UV did not provide enough sensitivity, as the sample amount injected into the system was very low. For this reason, the LLE and SPE experiments were needed prior to the HPLC detection.

TDAs and MDAs have two functional groups (-NH_2_) that are polar and partly soluble in water; as a result, the extraction of these compounds from aqueous solution is difficult [11].

TDAs (single ring) in comparison with MDAs (two rings) have more polarity and are more soluble in water. Therefore, the mean recoveries for the extraction of TDA derivatives (2,4-TDA and 2,6-TDA) should be less than that of MDA derivatives (4,4′-MDA, 2,4′-MDA, and 2,2′-MDA).

In liquid extraction methods, in general data obtained for mean recoveries of extraction for both LLE and DLLME were not very satisfactory. The mean recoveries of LLE is better than those of DLLME, because the number of extraction steps could be increased in the LLE method, while there is just one time dispersion in the DLLME method. The mean recoveries for the extraction of MDAs were greater than that of TDAs in both methods. DLLME were used in extraction of aromatic amines in aqueous matrix with acceptable recoveries [8, 9], but in our research, the interested aromatic amines have two functional group (-NH_2_) which are more soluble in aqueous solutions, and consequently, recoveries are lower than LLE and other methods.

**Figure 1:**
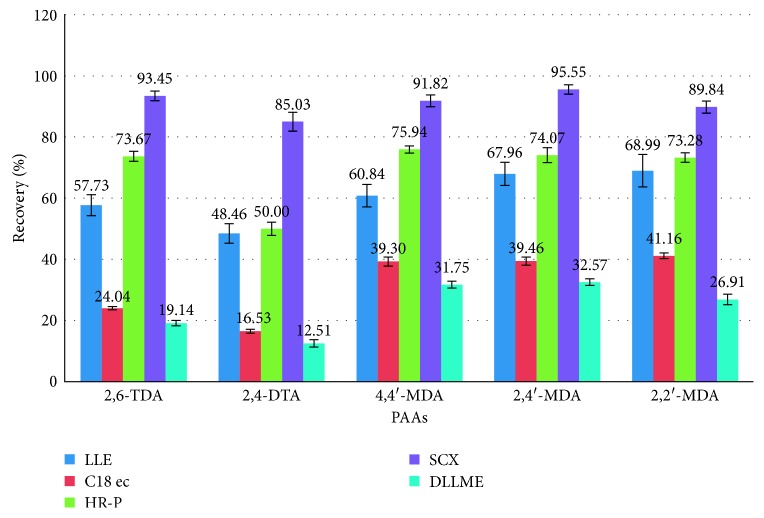
Compressive of recoveries of extraction methods: LLE, DLLME, SPE C18 ec, SPE HR-P, and SPE SCX for 5 primary aromatic amines.

In SPE methods, the mean recoveries of TDAs and MDAs were in the order of SCX > HR-P > C18 ec. Octadecyl sorbents (nonpolar) are not suitable for TDAs and MDAs in the C18 cartridge. In HR-P columns, the cross-linking in SDVB leads to better adsorption than C18 for the polar PAAs. The mean recoveries of the five extraction methods were compared, and the SPE method with SCX cartridge was the best (more than 90%). The result of all applied methods are compared and shown in Figure 1.

**Figure 2:**
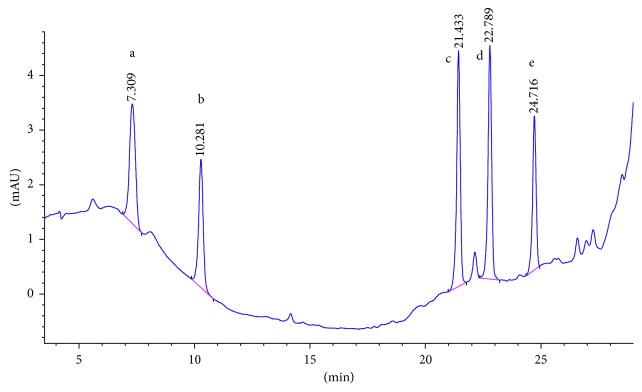
Chromatogram of the standard solution (100 ppb). Peak identified as a: 2,6-TDA, b: 2,4-TDA, c: 4,4′-MDA, d: 2,4′-MDA, and e: 2,2′-MDA.

**Figure 3:**
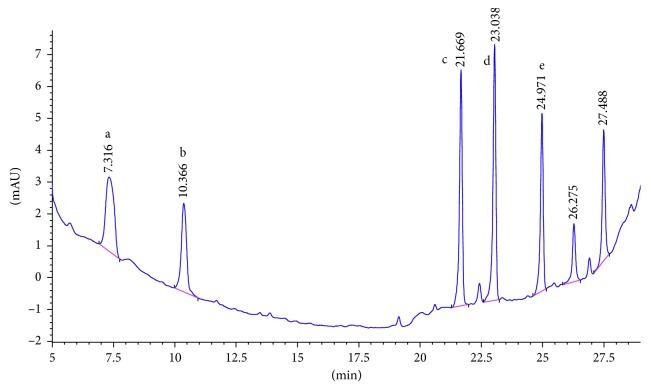
Chromatogram of extracted sample spiked (15 ppb) in 3% acetic acid with SPE SCX. Peak identified as a: 2,6-TDA, b: 2,4-TDA, c: 4,4′-MDA, d: 2,4′-MDA, and e: 2,2′-MDA.

**Figure 4:**
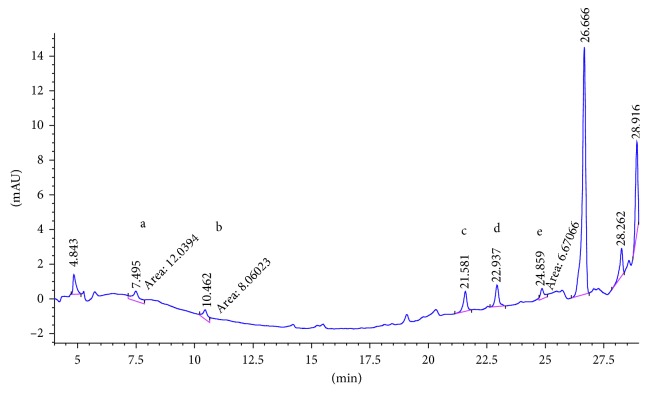
Chromatogram of extracted sample spiked (15 ppb) in 3% acetic acid with LLDME. Peak identified as a: 2,6-TDA, b: 2,4-TDA, c: 4,4′-MDA, d: 2,4′-MDA, and e: 2,2′-MDA.

**Figure 5:**
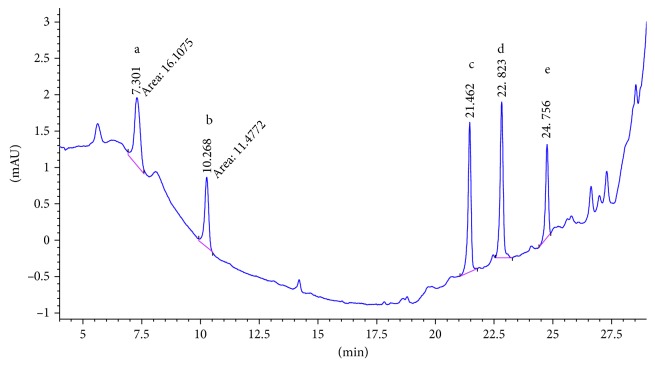
Chromatogram of extracted sample spiked (15 ppb) in 3% acetic acid with SPE C18. Peak identified as a: 2,6-TDA, b: 2,4-TDA, c: 4,4′-MDA, d: 2,4′-MDA, and e: 2,2′-MDA.

**Figure 6:**
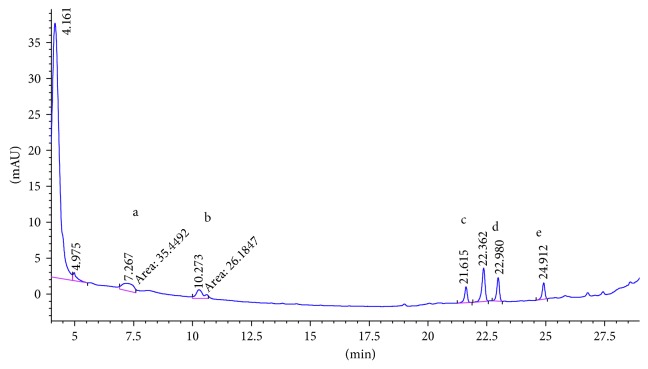
Chromatogram of extracted sample spiked (15 ppb) in 3% acetic acid with SPE HR-P. Peak identified as a: 2,6-TDA, b: 2,4-TDA, c: 4,4′-MDA, d: 2,4′-MDA, and e: 2,2′-MDA.

**Figure 7:**
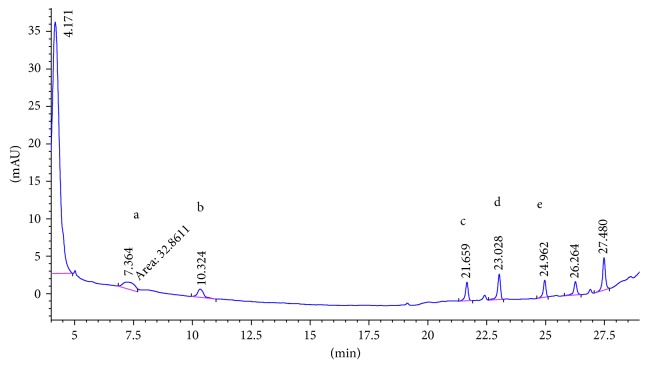
Chromatogram of extracted sample spiked (15 ppb) in 3% acetic acid with LLE. Peak identified as a: 2,6-TDA, b: 2,4-TDA, c: 4,4′-MDA, d: 2,4′-MDA, and e: 2,2′-MDA.

SCX cartridge with the mechanism of action being based on ion exchange could properly adsorb polar PAAs and so has the best mean recovery among the studied cartridges.  Figure 2 shows the chromatogram of the standard solution of five PAAs (100 ng·ml^−1^). The chromatogram of 15 ng·ml^−1^ spiked food simulant, in which the final concentration of the extracted and preconcentrated solution by SCX cartridge is 150 ng·ml^−1^, is shown in Figure 3. Also the chromatograms of each method are compared for the performance of the extraction and shown in Figures 4–7.

HPLC method combined with SCX cartridge was validated and developed for the separation and quantitation of five primary aromatic amines in 3% acetic acid as food simulant. After optimizing all separation parameters, the good separation of five PAAs in food simulant was feasible within 30 min. Additionally, the method was validated for linearity, LOD, precision, and intra- and interday variation.

## 4. Conclusions

It is important to determine and control the migration of contaminants from materials in contact with food into the food. Packaging materials provided this route for migration of contaminants such as PAAs from PU adhesives which are used in laminated multilayer films; some PAAs can migrate to the food. Analysis of PAAs is very valuable in the European regulations, and for this purpose, EU has established regulations for PAAs. Simultaneous extraction and analysis of PAAs (2,4-TDA, 2,6-TDA, 4,4′ MDA, 2,4′ MDA, and 2,2′ MDA) lead to the process of food safety assessment and maintenance, also a means to monitor the use of authorized adhesives and prevention of unauthorized ones being used. Solid-phase extraction SCX is introduced as a suitable method for extraction of polar primary aromatic amines (PAAs) which might have migrated from the laminated multilayer films of packaging materials into 3% acetic acid as food simulant.

## Figures and Tables

**Table 1:** Linearity of PAAs in 3% acetic acid.

Compounds	Regression equation	Linear range (mgl^−1^)	Correlation coefficient (*R*^2^)
2,6-TDA	*Y* = 0.438*X* − 2.152	0.05–0.8	0.9996
2,4-TDA	*Y* = 0.356*X* + 2.6455	0.05–0.8	0.9987
4,4′-MDA	*Y* = 0.5104*X* − 6.0336	0.05–0.8	0.9998
2,4′-MDA	*Y* = 0.4868*X* − 4.2338	0.05–0.8	0.9999
2,2′-MDA	*Y* = 0.3734*X* − 2.3448	0.05–0.8	0.9999

**Table 2:** The recovery of PAAs in 3% acetic acid.

Compounds	Amount spiked (*μ*g·l^−1^)	Recovery (%)	RSD	Amount spiked (*μ*g·l^−1^)	Recovery (%)	RSD	Amount spiked (*μ*g·l^−1^)	Recovery (%)	RSD
*First day*									
2,6-TDA	7.5	90.26	2	15	93.74	1.4	30	96.46	1.3
2,4-TDA	7.5	74.4	3.3	15	87.77	3.3	30	92.92	2.6
4,4′-MDA	7.5	83.94	2.6	15	95.28	0.95	30	96.33	0.95
2,4′-MDA	7.5	95	1.6	15	94.76	1.4	30	96.86	1.4
2,2′-MDA	7.5	81.58	2.69	15	93.58	2.71	30	94.79	1.1
*Second day*									
2,6-TDA	7.5	90.77	2.6	15	94.04	1.4	30	96.94	1.3
2,4-TDA	7.5	74.90	4.2	15	91.97	4.1	30	93.9	1.1
4,4′-MDA	7.5	89.26	2.7	15	94.53	1.3	30	97.03	0.43
2,4′-MDA	7.5	96.76	1.8	15	97.08	1.1	30	97.15	0.96
2,2′-MDA	7.5	85.02	2.18	15	93.16	2.07	30	94.93	4.6
*Third day*									
2,6-TDA	7.5	89.76	2.4	15	94.90	1.5	30	95.80	1.5
2,4-TDA	7.5	77.9	4.8	15	91.21	2.2	30	92.9	1.8
4,4′-MDA	7.5	88.13	0.94	15	94.79	2.5	30	96.29	0.4
2,4′-MDA	7.5	94.94	2.17	15	97.17	2.2	30	98.05	1.1
2,2′-MDA	7.5	85.38	3.37	15	94.82	4.6	30	95.44	0.61

**Table 3:** Intra- and interday precision of determination of PAAs in 3% acetic acid.

Compounds	Amount spiked (*μ*gl^−1^)	Precision (intraday) RSD	Precision (interday) RSD
2,6-TDA	75	0.115	0.146
	150	0.115	0.278
	300	0.2	0.239

2,4-TDA	75	0.115	0.188
	150	0.173	0.161
	300	0.152	0.215

4,4′-MDA	75	0.058	0.139
	150	0.152	0.273
	300	0.1	0.256

2,4′-MDA	75	0.115	0.115
	150	0.404	0.292
	300	0.058	0.153

2,2′-MDA	75	0.1	0.086
	150	0.152	0.165
	300	0.1	0.1

**Table 4:** LOD and LOQ of the method of determination of PAAs in 3% acetic acid.

Compounds	Limit of detection (*μ*gl^−1^)	Limit of quantitation (*μ*gl^−1^)	RSD
2,6-TDA	1.88	5.71	0.250
2,4-TDA	1.86	5.64	0.201
4,4′-MDA	1.44	4.37	0.223
2,4′-MDA	1.36	4.13	0.201
2,2′-MDA	2	6.08	0.227

## Abbreviations

DLLME: Dispersive liquid-liquid microextraction
HPLC-UV: HPLC-ultraviolet detector
LLE: Liquid-liquid extraction
MDA: Methylenedianiline
MDI: Methylene diphenyl isocyanate
PAAs: Primary aromatic amines
PU: Polyurethane
SPE: Solid-phase extraction
TDA: Toluenediamine
TDI: Toluene diisocyanate.
