# Erratum to “Comparison between Different Extraction Methods for Determination of Primary Aromatic Amines in Food Simulant”

**DOI:** 10.1155/2018/6423428

**Published:** 2018-09-06

**Authors:** Morteza Shahrestani, Mohammad Saber Tehrani, Shahram Shoeibi, Parviz Aberoomand Azar, Syed Waqif Husain

**Affiliations:** ^1^Department of Analytical Chemistry, Faculty of Basic Science, Science and Research Branch, Islamic Azad University, Tehran, Iran; ^2^Food and Drug Laboratories Research Center (FDLRC), Iran Food and Drug Administration (IFDA), MOH, Tehran, Iran

In the article titled “Comparison between Different Extraction Methods for Determination of Primary Aromatic Amines in Food Simulant” [1], there was an error in the affiliations' list, where the second, third, and fourth affiliations should be removed. This error occurred in the production process. The updated affiliations' list is shown above.
